# Editorial: The Role of Steroid Hormones and Growth Factors in Cancer

**DOI:** 10.3389/fcell.2022.887529

**Published:** 2022-04-12

**Authors:** Gustavo Cernera, Marzia Di Donato, Paul J. Higgins, Isabel R. Schlaepfer

**Affiliations:** ^1^ CEINGE, Advanced Biotechnologies, Naples, Italy; ^2^ Department of Molecular Medicine and Medical Biotechnologies, University of Naples Federico II, Naples, Italy; ^3^ Department of Precision Medicine, University of Campania “L Vanvitelli”, Naples, Italy; ^4^ Department of Regenerative and Cancer Cell Biology, Albany Medical College, Albany, NY, United States; ^5^ Division of Medical Oncology, Genitourinary Cancer Program, School of Medicine, University of Colorado, Aurora, CO, United States

**Keywords:** steroid hormones and receptors, growth factors, cancer biology, cancer cells, therapeutic breakthroughs

The relationship between steroid hormones (SHs), growth factors (GFs), their cognate receptors and the downstream signaling pathways lie at the center of cancer development, progression, and therapeutic resistance (Gao et al., 2002; Witsch et al., 2010). A plethora of direct and indirect mechanisms have been described that link SHs- and GFs-signaling to carcinogenesis; nonetheless, the complete picture remains unclear and the underlying mechanisms uncertain. The collection of papers in this Special Issue demonstrates the complexity of issues in this field and provides an update on the latest findings regarding SHs and GFs in cancer, with a focus on future therapeutic breakthroughs. Cancer was associated with soluble GFs for the first time in the 1950s (Waterfield et al., 1983). Later studies established that cells derived from different human tumors are not only stimulated by GFs, but they can also release GFs for an autocrine-regulation of cell proliferation and migration (Sporn and Roberts, 1985). In this context, high plasma levels of Insulin-like growth factor-1 (IGF-1) represent a risk factor for the development and recurrence of breast cancer (BC) in the general population and its receptor, IGF-IR, is overexpressed and hyper-phosphorylated in several subtypes of BCs. In this Special Issue, Lanza et al. describe in their interesting review the pathway underlying IGF-1/IGF-IR signaling and their co-protagonists that lead them to propose combinatorial therapies. In parallel, reducing the IGF1 circulating levels with dietary restrictions could exert anticancer effects by promoting apoptosis, inhibiting angiogenesis, and impairing the downstream engagement of the IGF1/IGF1R pathway. On this topic and with a broader discussion about dietary energy modulation and autophagy, nutritional deprivation of tumor cells as a therapeutic strategy is the subjected of the review by Cozzo et al. The insulin and insulin-like growth factor system (IIGFs) and estrogenic signaling intersect and have high impact in modulating the crosstalk between BC cells and its tumor microenvironment. The review by Vella et al. nicely describes how estrogen and the IIGFs impact stromal elements through soluble and non-soluble secreted molecules, which regulate ECM remodeling, neoangiogenesis, migration, and invasion. The authors open new perspectives: targeting the estrogen–IIGFs cross-talk in both cancer cells and the tumor microenvironment may well be an effective therapeutic option, particularly in patients affected by hyperinsulinemia due to insulin resistance.

BC is an eterogeneous disease and effective therapies are elusive for the more aggressive subtype, triple negative BC (TNBC). By using cellular and animal models, Du et al. describe the role of STAT3 in proliferation and invasiveness of MA-891 cells and the growth of TA2 xenografts and propose STAT3 as a potential therapeutic target for patients affected by TNBC. Data presented by Di Donato et al. show that TNBC cells, expressing significant amounts of TrkA, release abundant quantities of biologically active Nerve Growth factor (NGF). NGF, through the TrkA/β1-integrin/FAK/Src complex, induces mitogenesis, cell migration and increases in multicellular spheroid size and the associated extracellular matrix (ECM). Similarly, as described by Cheaito et al., epidermal growth factor (EGF) is determinant for the growth of different patient-derived prostate cancer (PC) cells from 3D-organoids. GFs are short-range mediators, which can be exchanged between cancer cells and ECM (Lee et al., 1984) stromal cells [such as tumor microenvironment cells; (Kalluri, 2016; Di Donato et al., 2021)], non-malignant cells and inflammatory cells (Zamarron and Chen, 2011). This cross-talk has a key role in tumor progression (Witsch et al., 2010), angiogenesis (Ucuzian et al., 2010) and inflammation (Wahl et al., 1989). Carcinoma-associated fibroblasts (CAFs) are an heterogeneous and dynamic population that play a major role in the initiation and progression of variousl malignancies by remodeling the supporting stromal matrix into a dense, fibrotic structure while secreting factors that lead to the acquisition of cancer stem-like characteristics. CAFs communicate via autocrine or paracrine mechanisms as well as by release of extracellular vescicles (EVs) with other cellular types in the tumor microenvironment and their secretoma are a valuable source of biomarkers to improve patient selection and treatment follow-up. These and other CAFs attributes are described in Linares et al. intriguing review.

In this context, *fibroblast growth factor 23* (FGF23) is highly expressed in osteomalacia (TIO) and in the oncogenic hypophosphatemic TIO, but also detactable in other types of cancer. By activating the FGF receptor 1c/αKlotho, FGF23 promotes the bone-like microenvironment in phosphaturic mesenchymal tumor, mixed connective tissue variant (PMTMCT), enhancing the FGF23 production by the tumor cells and worsening TIO as assessed by Ewendt et al. In osteosarcoma (OS), the safe application of Bone Morphogenic Protein-2 (BMP-2) in clinical settings remains unclear. In their review, Xu et al. propose a low-dose and slow-release strategy of BMP-2 for bone regeneration protocols. They suggest reconsidering BMP-1 use in patients with bone metabolic diseases, since it might increase (if used at supra-physiological concentration) the occurrence of OS. It is important in this regard to remember that SHs and GFs signaling are often cooperative. In this regard, Bleach et al. describe the intersection between the IGF/IGF-1R axis and different SHs evident in normal growth and development, and extending to metabolism disorders and various endocrine-related cancers. The authors analyze this cross-talk also taking in consideration clinical trials targeting IGF in cancer. SHs have pivotal roles in the common hormone-dependent types of cancer, such as BC and PC. By using T-47D human BC cells, Magali Mondaca et al., demonstrate that after luteinizing hormone (LH) stimulation, the receptor LHR, trough non-genomic actions, recruites and activates a signalling cascade involving Src, FAK and paxillin, leading to an increased phosphorylation and translocation of N-WASP, which culminates in cell migration, invasion and cytoskeletal reorganization. Considering the available data in literature and analyses of the TCGA database, Orzechowska et al. analyzed genes responsive to Notch signaling in BC and in other female reproductive tract tissues, including ovary, cervix, and uterine endometrium and confirmed distinct expression profiles of Notch pathway members as well as their target genes in normal tissues compared their cancerous counterparts. Searching for new therapeutic targets based on specific Notch pathway profiles may be a promising strategy.

PC is the second leading cause of death in men and androgen-deprivation therapy (ADT) is the first treatment option. However, as detailed in the manuscript presented by Feng et al., this type of therapy is not without adverse effects. When ADT is employed for patients with non-metastatic localized PC an increase of fatigue is common and likely due to sleep-related impairment connected to alterations in steroid hormone biosynthesis.

The Androgen Receptor (AR) can interact with different proteins and its signaling in PC initiation or progression can be regulated by many non-coding RNAs that Yang et al. discuss as possible diagnostic biomarkers and therapeutic targets. Genetic rearrangements can promote the formation of fusion genes in which an androgen-regulated promoter is fused to a protein-coding area of a previously androgen-unaffected gene. The presence of these gene fusions is more frequent in PC than in other types of cancer. The role of pseudogenes and non-coding RNAs (ncRNAs), including long non-coding RNAs (lncRNAs), is the subject of the review by Scaravilli et al. A more troubling form of PC is the castration-resistant PC (CRPC), which occurs when PC develop resistance after first line therapies. Uo et al. shed light on mechanisms underlying the transition to a more metabolically aggressive PC phenotype. They explain in their review that two different metabolic and cellular adaptations are involved in PC progression. In the final stage, PC cells appear highly glycolytic, as determined by imaging with ^18^F-fluorodoxyglucose (^18^F-FDG), a tracer frequently used to assess tumor energetics. In addition, PC acquires a neuroendocrine phenotyope characterized by robust ^18^F-FDG uptake and loss of AR signaling. At this stage, unfortunately, the commonly used therapies fail, but in the recent years novel compounds, using alternative approaches to target the AR pathway directly or indirectly, are available. Among them, as described in the intriguing review by Obinata et al., BET inhibitors targeting BET proteins that directly interact with the N-terminal domanin (NTD) of AR, or OCT1 targeting molecules, seem to be efficacious. The authors propose an in-depth discussion about CRPC. The expression and the utility of assessing single nucleotide variants of AR and KIT genes were also investigated in Mexican patients with isolated Cryptorchidism (CO) and testicular germ cell tumors (TGCT). By using this approach Landero-Huerta et al. identified clinical features and genetic variants that may support the early diagnosis of TGCT in pediatric patients with isolated CO.

The paper by O’ Connell et al. describes the involvement of the AR in thyroid cancer where it inhibits NF-kB signaling and, by increasing the IkBα inhibitory subunit, negatively modulates the expression of the immune checkpoint molecule PD-L1. The AR has a recognized role also in BC. In this context, Bandini et al., propose the miRNA, miR-9-5p, as an inhibitor of AR expression, leading to an inverse correlation between miR-9-5p and AR in primary BC samples. The use of miR-9-5p inhibits proliferation of BC cells and revert the AR-downstream signaling. Dong et al. further describe in their paper, that miR-181d-5p negatively regulates Core 1 β 1, 3-galactosyltransferase 1 (C1GALT1), an enzyme highly expressed in lung adenocarcinoma (LUAD) and which promotes cell proliferation, migration, and invasion as well as tumor formation *in vivo*. In addition, they found a role for the axis miR-181d-5p/C1GALT1/RAC1 in the LUAD progression. Targeting C1GALT1 could be a potential therapeutic approach for fighting LUAD.

In conclusion, in this Special issue, we have invited leading research groups to contribute with Reviews and original Articles to enriche the knowledge on the role of SHs and GRs in cancer. The manuscripts here collected address some of the questions still pending and identify new challenges in this intricate field. It this the time to re-examine the molecular landscape of SHs and GFs and the possible intersection points in their signaling pathways and to consider the emerging molecular targets and the new drugs available.
